# Usefulness of minimally invasive autopsy in the diagnosis of arboviruses to increase the sensitivity of the Epidemiological Surveillance System in Ceará, Brazil

**DOI:** 10.1590/S2237-96222024V33E2024008.en

**Published:** 2024-05-24

**Authors:** Livia Mendes de Almeida, Deborah Nunes de Melo, Manuella Mendonça da Silva, Pedro Mansueto Melo de Souza, Fernanda Kézia de Sousa Silva, Tania Mara Silva Coelho, Shirlene Telmos Silva de Lima, Anacelia Gomes de Matos Mota, Renata Aparecida de Almeida Monteiro, Paulo Hilario Nascimento Saldiva, Geraldo Gileno de Sá Oliveira, Luciano Pamplona de Góes Cavalcanti

**Affiliations:** 1 Centro Universitário Christus, Faculdade de Medicina, Fortaleza, CE, Brazil; 2 Universidade Federal do Ceará, Programa de Pós-Graduação em Patologia, Fortaleza, CE, Brazil; 3 Secretaria da Saúde do Estado do Ceará, Serviço de Verificação de Óbito Dr. Rocha Furtado, Fortaleza, CE, Brazil; 4 Universidade Federal do Ceará, Ambulatório de Pesquisa Clínica Replick, Fortaleza, CE, Brazil; 5 Secretaria da Saúde do Estado do Ceará, Fortaleza, CE, Brazil; 6 Universidade Federal do Ceará, Programa de Pós-Graduação em Saúde Coletiva, Fortaleza, CE, Brazil; 7 Laboratório Central de Saúde Pública do Ceará, Fortaleza, CE, Brazil; 8 Universidade de São Paulo, Faculdade de Medicina de São Paulo, São Paulo, SP, Brazil; 9 Fundação Oswaldo Cruz, Centro Gonçalo Moniz, Salvador, BA, Brazil

**Keywords:** Arbovirus Infections, Autopsy, Epidemiologic Surveillance Services, Investigative Techniques, Infecciones por Arbovirus, Autopsia, Servicios de Vigilancia Epidemiológica, Técnicas de Investigación, Infecções por Arbovirus, Autópsia, Serviços de Vigilância Epidemiológica, Técnicas de Pesquisa

## Abstract

**Objective:**

To create a protocol for performing minimally invasive autopsies (MIA) in detecting deaths from arboviruses and report preliminary data from its application in Ceará state, Brazil.

**Methods:**

Training was provided to medical pathologists on MIA.

**Results:**

A protocol was established for performing MIA, defining criteria for sample collection, storage methods, and diagnoses to be carried out according to the type of biological sample; 43 MIAs were performed in three months. Of these, 21 (48.8%) arrived at the Death Verification Service (SVO) with arboviruses as a diagnostic hypothesis, and seven (16.3%) were confirmed (six chikungunya cases and one dengue case); cases of COVID-19 (n = 9), tuberculosis (n = 5), meningitis (n = 4), cryptococcosis (n = 1), Creutzfeldt-Jakob disease (n = 1), breast cancer (n = 1), and human rabies (n = 1) were also confirmed.

**Conclusion:**

The protocol implemented enabled identification of a larger number of suspected arbovirus-related deaths, as well as confirmation of other diseases of interest for surveillance.

## Study contributions

**Main results:**

A protocol was developed to perform minimally invasive autopsies (MIAs) in Death Verification Services (SVO), capable of expanding the system’s capacity to identify a greater number of deaths suspected to be due to arboviruses.

**Implications for services:**

The experience suggests that in-service trained health professionals are able to perform MIA, and that use of this technique in SVOs has been shown to be capable of increasing the system’s sensitivity in detecting deaths of interest to public health.

**Perspectives:**

Trained professionals will be able to collect biological material in hospitals, through MIA, in cases of interest for health surveillance and when family members do not allow a complete conventional autopsy to be performed.

## Resumen

**Objetivo:**

Estabelecer un protocolo utilizado para la realización de autopsias mínimamente invasivas (AMI) para la detección de muertes por arbovirus y presentar datos preliminares de este protocolo en Ceará, Brasil.

**Métodos:**

Se llevó a cabo la capacitación de médicos patólogos en AMI.

**Resultados:**

Se estableció un protocolo para la realización de AMI, que define los criterios para la toma de muestras, métodos de almacenamiento y diagnóstico; en tres meses se realizaron 43 AMI; de estas, 21 (48,8%) llegaron al Servicio de Verificación de Óbito (SVO) con una hipótesis diagnóstica de alguna arbovirosis y siete (16,3%) fueron confirmadas (seis casos de chikungunya y uno de dengue); también se confirmaron casos de Covid-19 (n = 9), tuberculosis (n = 5), meningitis (n = 4), criptococosis (n = 1), enfermedad de Creutzfeldt-Jakob (n = 1), neoplasia de mama (n = 1) y rabia humana (n = 1).

**Conclusión:**

El protocolo implementado permitió la identificación de un mayor número de muertes sospechosas de arbovirus, además de la confirmación de otras patologías de interés.

## Resumo

**Objetivo:**

Estabelecer protocolo para realização de autópsias minimamente invasivas (AMIs) na detecção de óbitos por arboviroses e relatar dados preliminares desse protocolo no Ceará, Brasil.

**Métodos:**

Médicos patologistas foram treinados em AMI.

**Resultados:**

Estabeleceu-se protocolo para AMI, definindo-se critérios para amostras a serem coletadas, suas formas de armazenamento e diagnóstico, segundo o tipo de amostra biológica; em três meses, foram realizadas 43 AMIs, das quais 21 (48,8%) chegaram ao Serviço de Verificação de Óbito (SVO) com hipótese diagnóstica de alguma arbovirose e sete (16,3%) foram confirmados (seis de chikungunya; uma de dengue); também foram confirmados casos de covid-19 (n = 9), tuberculose (n = 5), meningite (n = 4), criptococose (n = 1), doença de Creutzfeldt-Jakob (n = 1), neoplasia de mama (n = 1) e raiva humana (n = 1).

**Conclusão:**

O protocolo implantado permitiu a captação de um maior número de óbitos suspeitos de arboviroses, além da confirmação de outras patologias de interesse da vigilância.

## INTRODUCTION

Concerned with the arbovirus scenario in Brazil,^1^ ^)-(^ ^3^ the country’s Health Ministry recommends that deaths suspected to be due to this condition be investigated.^4^ Determining the cause of these deaths continues to be a challenge.^5^ Autopsy undoubtedly contributes to improving understanding of how microorganisms cause diseases, especially emerging and re-emerging diseases.^6^

Autopsies performed by the Death Verification Service (*Serviço de Verificação de Óbito* - SVO) of the Ceará State Health Department contributed to greater detection of deaths due to dengue, being one of the largest sources of notification of suspected deaths due to dengue in 2011-2012 and chikungunya in 2016-2017 in its territory.^7^ ^)-(^ ^10^

However, refusal to authorize autopsies is considerable, due to lack of information, prejudice or pressure from burial services.^11^ ^),(^ ^12^ Therefore, there is greater need to use safer and less invasive techniques to obtain organ samples for post-mortem analysis, potentially more acceptable to relatives or guardians of those who died.^13^ Another aspect to be mentioned about the issue was the emergence of the COVID-19 pandemic, which led most services to suspend autopsies for safety reasons.^14^ ^)-(^ ^16^

The objective of autopsies is to obtain more information about the pathological processes and determine the factors contributing to death.^17^ If, on the one hand, this practice has become increasingly difficult in cases in which the family does not allow it to be performed,^11^ on the other hand minimally invasive autopsy (MIA) has become an increasingly used method for collecting samples from key post-mortem organs.^12^ ^),(^ ^17^ ^)-(^ ^19^ MIA is a relatively simple technique for collecting tissue samples from various organs and body fluids; a quick, non-disfiguring procedure, easy to perform and capable of providing robust data for health surveillance.^20^ ^)-(^ ^23^

The objective of this study was to establish a protocol for performing MIAs in detecting deaths due to arboviruses, and to report preliminary data on its implementation in the state of Ceará, Brazil.

## METHODS

This was a report of an experience developed in partnership between the *Universidade Federal do Ceará* Pathology and Public Health Postgraduate Programs, the Ceará State Health Department, the Dr. Rocha Furtado Death Verification Service (SVO), the Faculty of Medicine of the *Centro Universitário Christus* and the Ceará Central Public Health Laboratory (*Laboratório Central de Saúde Pública* - LACEN/CE), as well as the *Hospital Clínic de Barcelona* Department of Anatomic Pathology and Microbiology and the *Instituto de Salud Global de Barcelona* (ISGlobal), Spain, to implement the MIA technique in Ceará.

The project subsequently received funding from the Brazilian Health Ministry, which, among other items, paid for the team of pathologists from Spain to come to Brazil to provide in-service training at the Dr. Rocha Furtado SVO. However, the onset of the COVID-19 pandemic led to the closure of Fortaleza’s international airport; subsequently, autopsies were suspended in accordance with Ministry of Health guidelines.^17^ At that time of pandemic crisis, the service’s need to implement MIA increased, given the growing number of corpses arriving at the SVO without an established underlying cause, whereby several of those deaths occurred at home.

**Figure 1:**
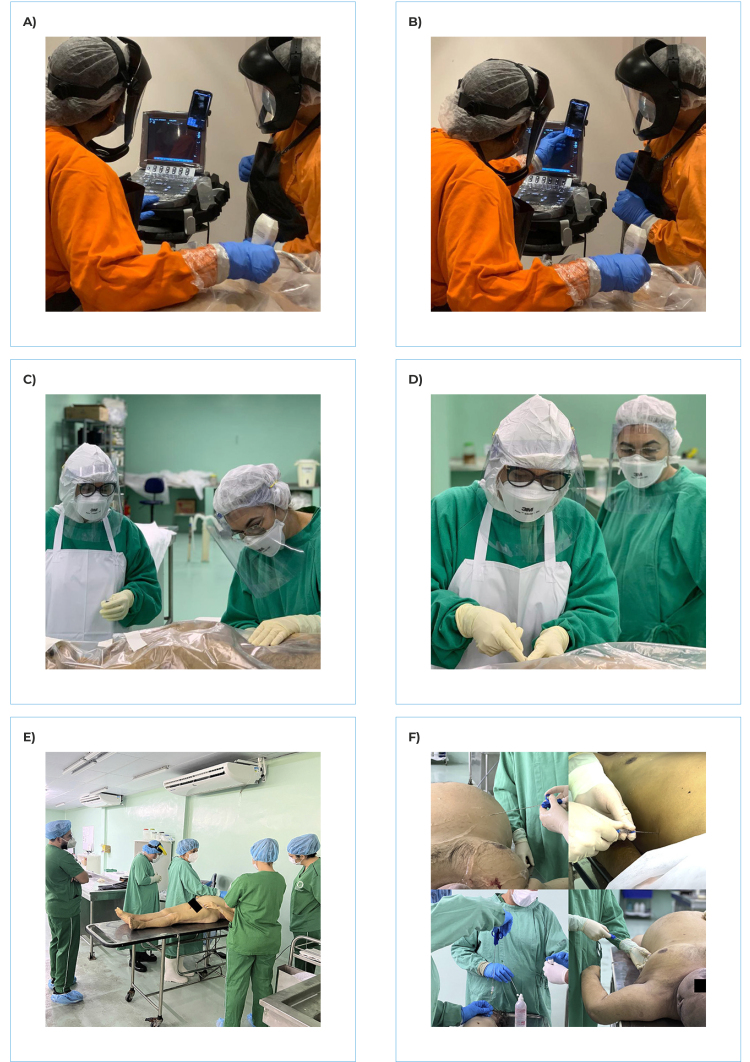
(A) training conducted on the Image Platform, *Universidade de São Paulo* Autopsy Room; (B) training conducted on the Image Platform, *Universidade de São Paulo* Autopsy Room; (C) performance of minimally invasive autopsy in Ceará; (D) performance of minimally invasive autopsy in Ceará; (E) performance of minimally invasive autopsy in Ceará; (F) performance of minimally invasive autopsy in Ceará

Given the urgent need to start using MIA, the Health Ministry facilitated negotiations between the Ceará group and the *Universidade de São Paulo* team to enable some Ceará pathologists to be trained. As part of this partnership, three professionals were sent to be trained in São Paulo (Figures 1A and 1B). The first experimental MIA in Ceará was performed in January 2021.^24^ As such the Ceará SVO was the second such service in Brazil to put this technique into practice (Figures 1C, 1D, 1E and 1F).

As the pandemic subsided, it was possible to carry out training at the Ceará SVO, between November 7^th^ and 11^th^, 2022, with the participation of and experience sharing between professionals from São Paulo, Barcelona/Spain, and the Health Ministry. Nine pathologists from Ceará received in-service MIA training. Once the local team had been trained, it was possible to establish work flows within the SVO and begin performing MIAs.

While the professionals were still considered to be in the training period, in those cases in which the family granted authorization, MIA was performed, followed by autopsy, with the aim of comparing the imaging findings with those of the biological samples sent to the reference laboratory. In those cases in which biological material was collected using both techniques, the procedures were performed by different pathologists. The result as to agreement between the two procedures occurred directly, by comparing the target organ macroscopic and microscopic findings.

The study project was approved by the *Centro Universitário Christus* Research Ethics Committee on February 20^th^ 2020, as per Certificate of Submission for Ethical Appraisal No. 27162619.1.0000.5049 and Opinion No. 3.851.684.

## RESULTS

During the first three months after in-service training, 43 MIAs were performed, of which 21 (48.8%) cases arrived at the SVO with arboviruses as a diagnostic hypothesis, while the remaining 22 (52.2%), were suspected cases of other conditions. Among the 21 deaths suspected to be due to arboviruses, seven (16.3%) were laboratory confirmed: six due to chikungunya and one due to dengue. The female sex predominated (79.2%) and average age was 54 years (< 1 to 100), with emphasis on the elderly (39% aged 70 or over).

Among the other conditions investigated, there were nine confirmed cases of COVID-19, five tuberculosis, four meningitis, one cryptococcosis, one Creutzfeldt-Jakob disease, one breast neoplasm and one human rabies case.

**Figure 2:**
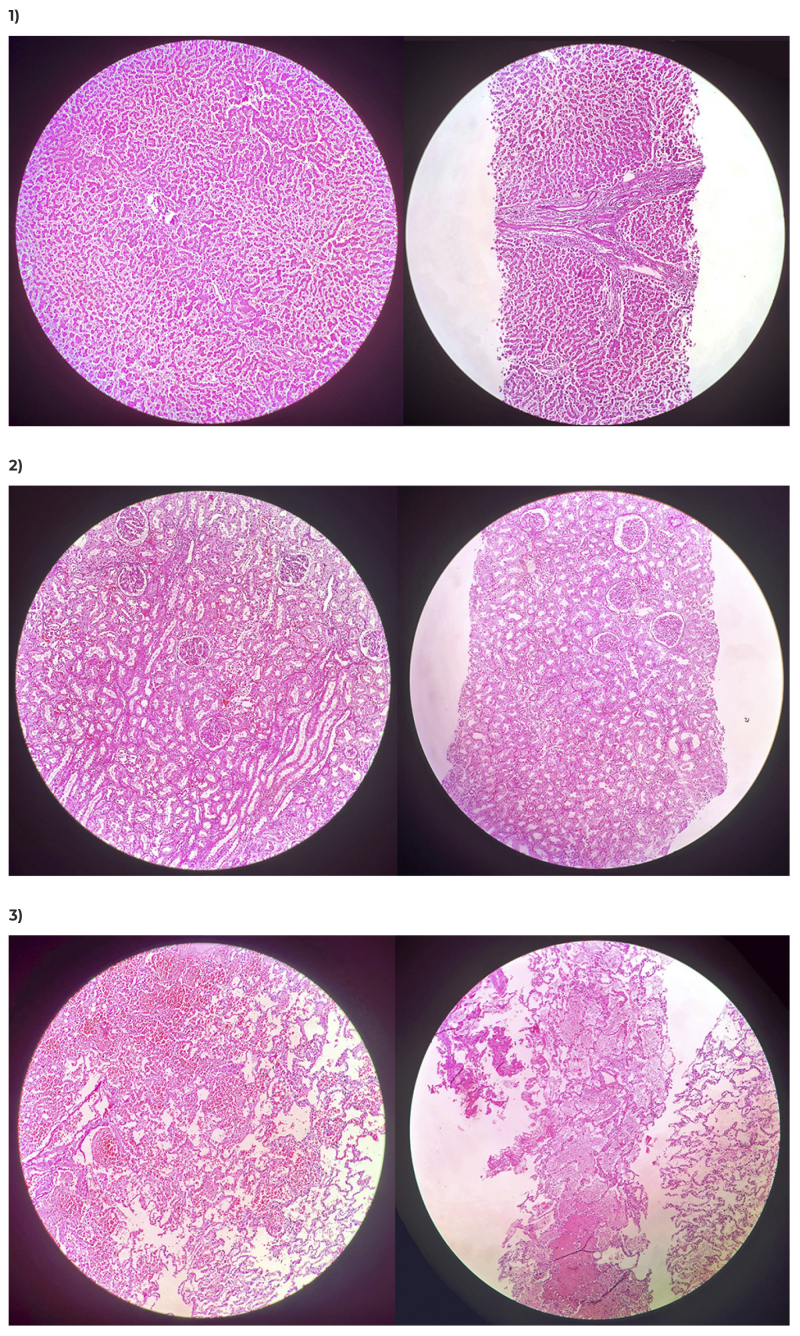
Comparison of histological sections of samples collected via autopsy (on the left) and via minimally invasive autopy (on the right) (Hematoxylin-Eosin, 100x)

Legend: 1) Hepatocyte disarrangement in liver samples; 2) Acute tubular necrosis in kidney samples; 3) Alveolar edema and hemorrhage in lung samples.

**Figure 3:**
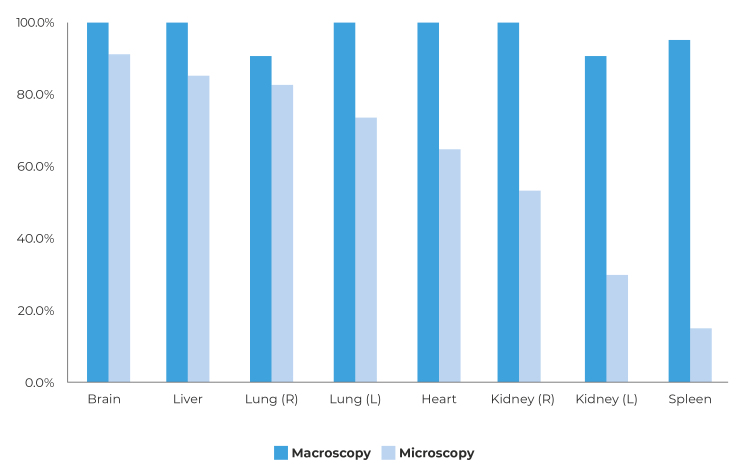
Percentage agreement between tissue collected via conventional autopsy and punctures via minimally invasive autopsies performed on the first 30 deaths investigated at the Ceará Death Verification Service (*Serviço de Verificação de Óbito* - SVO), January 29 - May 7, 2023

Notes: Macroscopy = when the pathologist was able to collect fragments that, macroscopically, appeared to be from the puncture target organ; Microscopy = when it was confirmed, microscopically, that the fragment collected via puncture corresponded to the puncture target organ.

Both techniques (MIA and conventional autopsy) were performed in 30/43 (60.7%) of the cases. The samples sent to the LACEN/CE laboratory had IgM and RT-qPCR positive results, both in blood, cerebrospinal fluid and viscera (brain and spleen) samples. Percentage agreement between the findings using the two techniques was greater for the brain, heart, lung and liver, and more complex for the spleen. It was possible to clearly identify findings such as liver hepatocyte disarrangement, acute kidney tubular necrosis, in addition to pulmonary alveolar edema and hemorrhage (Figures 2 and 3).

Based on this initial experience, a work flow was created and the necessary routines for carrying out MIA at the SVO were established, in addition to defining the following selection criteria:

a) death referred to the SVO with arbovirus as a diagnostic hypothesis; or

b) death referred to the SVO whereby suspicion of arbovirus is raised following interview with family members; or

c) death for which family members or guardians do not authorize conventional autopsy; or

d) death due to high-risk disease, whereby autopsy is contraindicated.

If MIA was indicated by any of these criteria, family member authorization was requested for performing minimally invasive autopsy, upon completion of an Informed Consent Form regarding the procedures involved. Once such authorization was received, the post-mortem study was begun using MIA, separating and identifying all the material to be used. Semi-automatic coaxial needles, of the TRU-CUT type, 16G x 20 cm long for adults and 14G x 9 cm long for children were used to perform MIA. Individual needles were used for each organ biopsied: brain, heart, right lung, left lung, liver, spleen, right and left kidneys, in that order.

The fragments collected using MIA were distributed for biomolecular, immunohistochemical and histopathological analyses, according to the following protocol:

- eight needles, with their respective eight guide wires, for puncturing the eight key organs (brain, heart, right lung, left lung, liver, spleen, right kidney and left kidney);

- ten formaldehyde-free cryotubes, for fresh packaging of blood, cerebrospinal fluid and eight key organ samples, to be sent for serological and molecular biology analysis at the LACEN/CE laboratory;

- eight cryotubes containing 10% buffered formaldehyde, for packaging the fragments of each of the eight key organs, to be sent for immunohistochemical analysis at the LACEN/CE outsourced reference laboratory;

- eight cryotubes containing 10% buffered formaldehyde, for packaging the fragments of each of the eight key organs, to be processed for histopathological analysis at the SVO itself.

After performing all MIA punctures, the corpses were transferred from the stretcher to the necropsy table and underwent autopsy, involving opening the cranial and thoracoabdominal cavities. Fragments from all key organs were also collected and each of them were fully examined, choosing the best sample from each organ for analysis.

Once MIA and the conventional autopsy had been completed, all samples were sent to the laboratories and their results were later compared in order to validate the technique regarding arbovirus cases.

## DISCUSSION

The creation and implementation of the protocol by the Ceará SVO enabled cases of deaths of interest to epidemiological surveillance in which the corpses did not have family authorization for performing a conventional autopsy to undergo the MIA technique, enabling the collection of biological material to investigate the cause of death. Use of MIA made it possible to increase both the number of suspect deaths to be investigated and also the sensitivity of the investigation system.

One limitation of the study that stands out is that, even though it is a safe, fast, accessible technique with greater acceptability, regardless of religious and ethical impediments,^25^ ^),(^ ^26^ use of MIA met with resistance from some pathologists. It is also worth mentioning the difficulty in identifying and puncturing small focal lesions, which affect only a small portion of a given organ. Focal lesions, such as nodules or abscesses, for example, may not be punctured if random punctures are performed. This limitation is partially resolved with the use of ultrasound, which enables identification of a large number of focal lesions, as well as enabling puncturing to be directed towards them. A further limitation relates to identifying focal lesions that do not show changes when using ultrasound, such as in areas of myocardial ischemia. For this type of lesion, however, there are no MIA techniques that satisfactorily replace the macroscopic analysis that conventional autopsy provides. Despite these limitations, it is worth highlighting that using MIA it is possible to identify the etiological agent in the majority of deaths due to infectious causes.^21^

Considering the primary objective of the protocol, i.e. to demonstrate the usefulness of MIA in diagnosing arboviruses, and given that it is known that arbovirus infections are systemic and affect organs in a diffuse manner, the limitations metioned above tend not to substantially jeopardize what was proposed. Even though it is not possible to identify focal lesions in the heart, for example, since myocarditis due to arbovirus is diffuse, the odds are high that this form of myocarditis can be detected by puncturing random portions of the myocardium.

It is important to make it clear that use of MIA should not be encouraged at the expense of performing autopsies. Autopsy remains the gold standard but, in cases in which there is no SVO or when the family does not authorize an autopsy, MIA is an alternative.

The first MIA in Brazil was performed in São Paulo, in March 2020,^27^ and later in the state of Ceará^24^ and then in the state of Bahia.^28^

Confirmation of a case of human rabies in Ceará, after several years with no record of the disease in the state, was evidence of the system’s increased sensitivity with the use of MIA: the family refused to authorize autopsy and the case would not have been investigated if MIA had not been performed at the hospital where the death occurred. This is, furthermore, an aspect to be discussed in this scenario of expanding the use of this technique to hospitals: i.e. whether or not MIA should be exclusively performed by pathologists. The experience of the *Universidade de São Paulo* points to the possibility of the procedure being performed by trained health professionals, even if they are not physicians. In Ceará, it was decided to only train pathologists, especially because it is they who have to provide histological diagnosis, as recommended by Brazilian legislation. In cases in which there is no SVO in the region, performance of MIA by other health professionals should be discussed and assessed. At the time of concluding this report, there is no consensus on the subject but it certainly deserves reflection, given the small number of pathologists available in the health system.

Even though the initial objective of the Ceará SVO was to use MIA in arbovirus cases, in a complementary way, indications for using MIA have been extended to other infectious diseases of public health concerrn, in order to increase the number of procedures performed by each pathologist, accelerating the learning curve of the technique and increasing sensitivity of death surveillance in Ceará.^29^

The SVO experience suggests that, when performing MIA, the corpse should be placed on a stretcher. Conventional necropsy tables have raised edges, which can make it difficult to puncture more dorsal structures, such as the kidneys. With the corpse in prone position, a suboccipital puncture is performed to collect cerebrospinal fluid from the cisterna magna. As there is no mandatory order for performing the procedure, it is recommended that each health service or professional should systemize it, so as to avoid forgetting to puncture one or more organ. The following puncture order has been established at the Ceará SVO: brain, heart, right lung, left lung, liver, spleen, right kidney and left kidney.

Regarding COVID-19 cases, it is worth mentioning that there are studies that report that corpses subjected to MIA had almost identical histological findings when compared to those subjected to autopsy.^30^

Initially, a needle was used for each organ due to the secondary objective of identifying which organ had positive laboratory results. Furthermore, the aim was to identify whether there was an organ with greater positivity than the others, with a view to prioritizing it when it was not possible to collect samples from all organs. This increases the cost of the procedure but, under normal conditions, needle consumption will be much lower.

There is no way of measuring the number of MIAs needed for a health professional to achieve a target organ success rate of 100%. The Ceará experience suggests that, with few autopsies performed, the professional has sufficient security and, if the service has ultrasound equipment and a radiologist, the MIA technique becomes more effective, facilitating completion of the process.

Historically, family members refusing to authorize autopsies has been greater in Ceará, which ends up motivating the search for less invasive alternatives; especially after the chikungunya epidemic, responsible for many deaths in elderly people whose relatives did not allow an autopsy to be performed.

Adopting MIA should not require new SVOs. Its use can expand the range of diseases observed through postmortem punctures, detection of emerging diseases and even diagnosis of chronic diseases.

The next challenge for the state of Ceará will be to train infectious disease specialists and neurologists from local reference hospitals - *Hospital São José de Ciências Infecciosas* and *Hospital Geral de Fortaleza*, both located in Ceará´s capital city - to use the technique in their hospitals, in cases that are of interest to health surveillance and, especially, in cases in which family members do not allow the corpse to be sent to the Death Verification Service. Greater use of minimally invasive autopsy will certainly contribute to reducing the number of deaths with ill-defined causes in Ceará.
